# Dengue virus pathogenesis and host molecular machineries

**DOI:** 10.1186/s12929-024-01030-9

**Published:** 2024-04-22

**Authors:** Saumya Sinha, Kinjal Singh, Y. S. Ravi Kumar, Riya Roy, Sushant Phadnis, Varsha Meena, Sankar Bhattacharyya, Bhupendra Verma

**Affiliations:** 1 Department of Biotechnology, All India Institute of Medical Sciences, Ansari Nagar, New Delhi, 110029 India; 2 Department of Biotechnology, M. S. Ramaiah Institute of Technology, MSR Nagar, Bengaluru, India; 3 Translational Health Science and Technology Institute, NCR Biotech Science Cluster, Faridabad, India

**Keywords:** Dengue virus, Epigenomic regulation, Transcription regulation, Post-transcriptional modifications, Translation regulation, Post-translational modifications, Stress granule formation

## Abstract

Dengue viruses (DENV) are positive-stranded RNA viruses belonging to the Flaviviridae family. DENV is the causative agent of dengue, the most rapidly spreading viral disease transmitted by mosquitoes. Each year, millions of people contract the virus through bites from infected female mosquitoes of the *Aedes* species. In the majority of individuals, the infection is asymptomatic, and the immune system successfully manages to control virus replication within a few days. Symptomatic individuals may present with a mild fever (Dengue fever or DF) that may or may not progress to a more critical disease termed Dengue hemorrhagic fever (DHF) or the fatal Dengue shock syndrome (DSS). In the absence of a universally accepted prophylactic vaccine or therapeutic drug, treatment is mostly restricted to supportive measures. Similar to many other viruses that induce acute illness, DENV has developed several ways to modulate host metabolism to create an environment conducive to genome replication and the dissemination of viral progeny. To search for new therapeutic options, understanding the underlying host-virus regulatory system involved in various biological processes of the viral life cycle is essential. This review aims to summarize the complex interaction between DENV and the host cellular machinery, comprising regulatory mechanisms at various molecular levels such as epigenetic modulation of the host genome, transcription of host genes, translation of viral and host mRNAs, post-transcriptional regulation of the host transcriptome, post-translational regulation of viral proteins, and pathways involved in protein degradation.

## Introduction

**Fig. 1:**
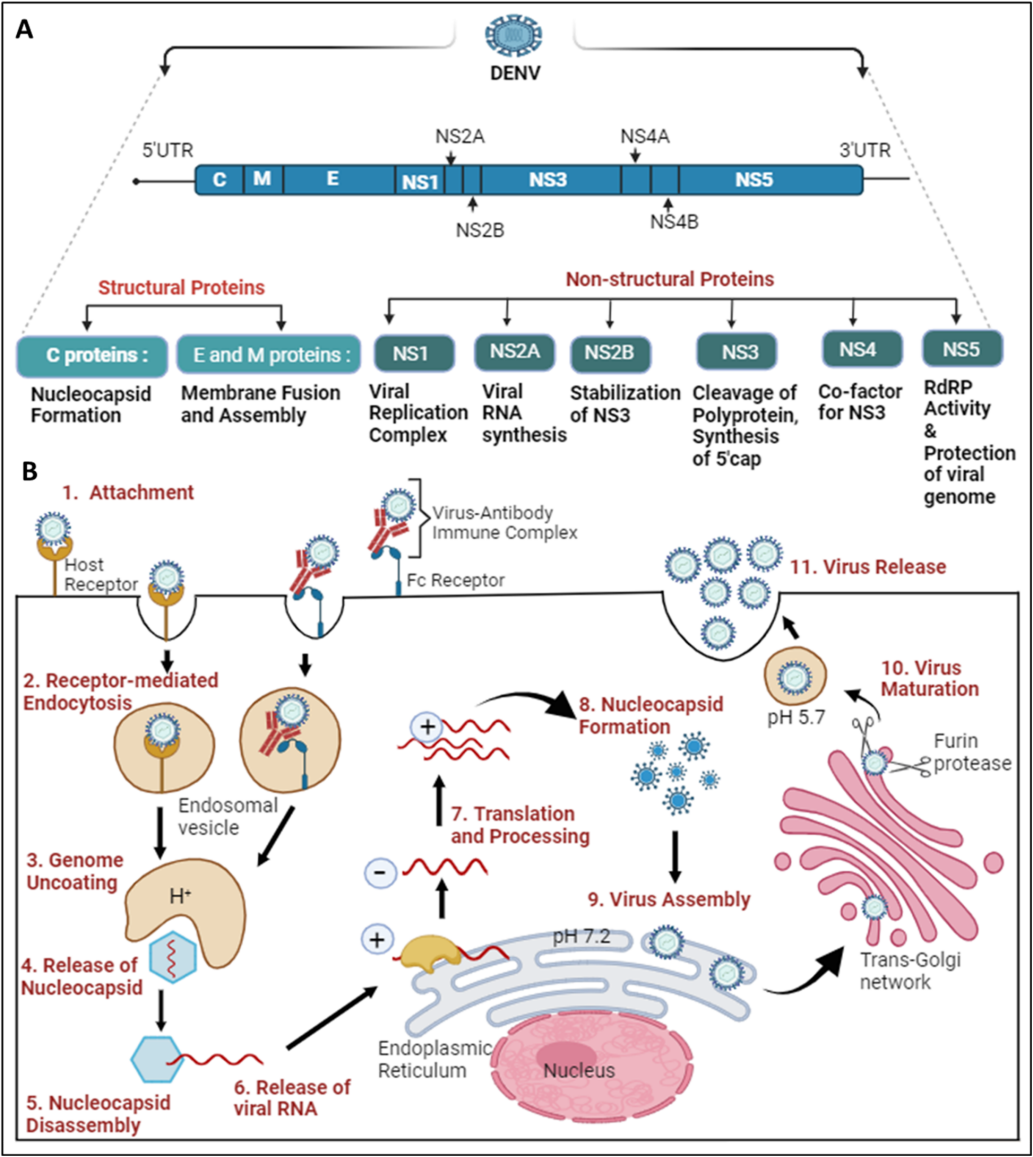
Structural organization and life cycle of DENV: **A** The Dengue virus genome comprises 5’UTR, ORF, and 3’UTR. The ORF translates into a polyprotein, which is further processed into three structural proteins: C (Capsid), E (Envelope), and M (Membrane), and seven non-structural proteins (NS1, NS2A, NS2B, NS3, NS4A, NS4B, NS5). **B** The initiation of the DENV replication cycle occurs with the entry into the cell via various host cell receptors or through the Fc region of the virus-antibody immune complex, which attaches to Fc receptors present on the target host cell. 1. DENV attaches to host cell receptors and enters the cell. 2. Internalization occurs through receptor-mediated endocytosis, forming an early endosome. 3, 4. Genome uncoating occurs as the pH decreases inside the early endosome; conformational changes take place, releasing the nucleocapsid into the cytoplasm. 5, 6. Disassembly of the nucleocapsid allows viral RNA assembly in the cytoplasm. 7. Viral RNA translocates into the Endoplasmic Reticulum, where translation results in a single polyprotein that is cleaved down by both host and viral proteases. Additionally, a translation switch results in the transcription of viral RNA employing antisense viral RNA. 8. The capsid protein encases the freshly created viral RNA to form the nucleocapsid. 9. Virus assembly occurs on the surface of the Endoplasmic Reticulum. 10. Immature viral particles are transported to the trans-Golgi network, where acidification results in conformational changes, followed by exposure to the furin protease to form mature viral particles. 11. Mature viral particles are exocytosed into the extracellular matrix, completing their replication cycle

Dengue virus (DENV), a positive-sense (+) single-stranded RNA virus belonging to the Flavivirus family, has spherical-shaped envelope virion particles with surface proteins arranged in icosahedral symmetry [1, 2]. The viral genome is about 11 kb long and is translated directly upon entering host cells by the protein synthesis machinery, producing both viral structural and non-structural proteins. The DENV genome synthesizes three structural proteins (Capsid, Membrane, Envelope) which form part of a mature virion particle, and seven non-structural proteins (NS1, NS2A, NS2B, NS3, NS4A, NS4B, and NS5), which helps in replication [3]. The RNA genome and the capsid protein interact to form a complex, while other structural proteins form part of the virion envelope [4]. Although the NS proteins are absent within the virion, they assist in virus replication and evasion of the immune system within an infected cell [3]. The structural organization and life cycle of DENV is shown in Fig. 1.

**Fig. 2:**
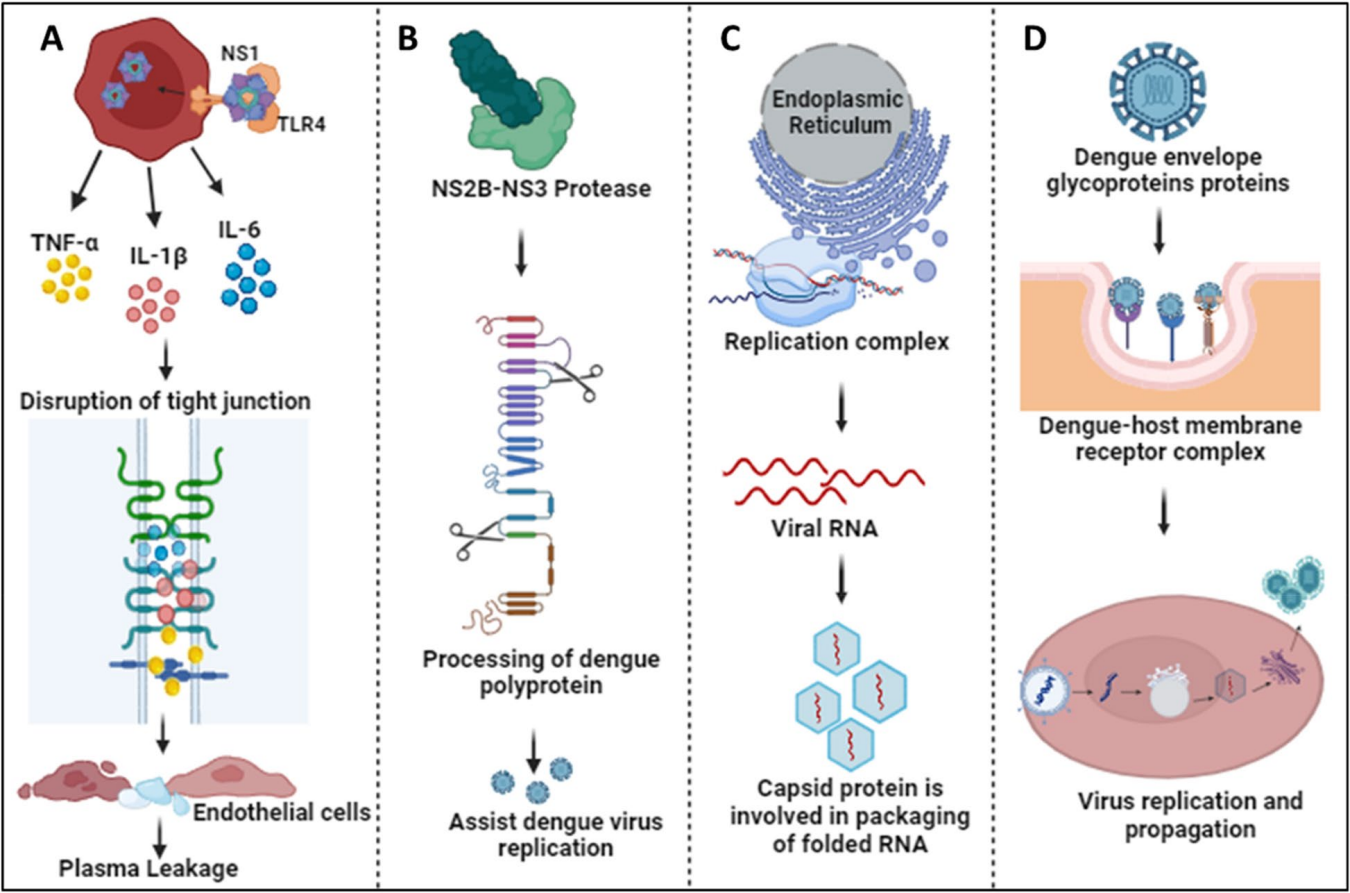
Physiological roles of DENV proteins: **A** The NS1 protein interacts with TLR4 and activates macrophages, resulting in cytokine release. Cytokines disrupt tight junctions and endothelial barriers, leading to plasma leakage in severe cases of dengue [8]. **B** NS3, together with the co-factor NS2B, participates in the processing of the dengue polyprotein, assisting in efficient virus replication [9]. **C** The Capsid protein helps in packaging folded RNA released from the DENV replication complex [4]. **D** Envelope glycoproteins attach to host receptors and form the dengue-host membrane complex, resulting in virus internalization to process its replication and propagation inside the host cell [13]

The NS1 protein has multiple roles inside and outside the host cell; it also serves as an early indicator to diagnose and assess the level of DENV infection [5, 6]. The protein is secreted as a hexamer into the blood circulation of patients, forming an open barrel structure with lipid molecules at the center [7]. NS1 plays a vital role in severe dengue physiopathology, specifically plasma leakage. A recent report suggested that NS1 activates macrophages via Toll-like receptor 4 (TLR4) and disrupts endothelial cells, resulting in vascular leakage as mentioned in Fig. 2A [8]. NS3 acts as a protease using NS2B as a co-factor, cleaving the polyprotein at specific sites and host proteins that would impair dengue infection as depicted in Fig. 2B [9]. The importance of the NS3 protein for the survival of the virus makes it a target for antiviral drugs as mentioned in Table 2. NS4B interacts with NS3 to modulate viral infection, while NS4A indirectly assists in the viral replication process by inducing autophagy, preventing cell death, which is beneficial for viral replication [10]. NS5 is the most conserved non-structural protein having an RNA-dependent RNA polymerase (RdRp) domain. NS5 is also involved in mRNA capping due to its methyltransferase and guanylyltransferase activities [11]. The NS5 protein is predominantly located in the nucleus of infected cells, believed to suppress the host anti-viral response [12]. The DENV envelope protein is involved in attachment and host cell receptor binding for virus entry into the host cell as mentioned in Fig. 2D [13]. The newly synthesized viral genome associates with the capsid to form a nucleocapsid, and it buds into the ER lumen together with viral E and prM proteins as shown in Fig. 2C [4].

Humans and mosquitoes are the primary hosts and vectors of DENV, respectively. The dengue virus is often undetectable yet prevalent, spreading in cycles of endemic and epidemic disease [14]. In human, the infection is spread by *Aedes* species of mosquitoes, specifically *Aedes aegypti, Aedes albopictus, and Aedes polynesiensis* [15]. In addition to infected female mosquitoes transmitting the virus to their offspring through eggs, mature female mosquitoes may become infected after feeding on the blood of DENV-infected patients. After ingestion by a mosquito, the virus infects the midgut followed by propagation to other tissues, including the salivary glands. When a mosquito feeds on blood, infectious virus is injected into the skin via salivary gland secretion. Soon after the virus enters the human host’s skin, it initiate its propagation and infects the resident cells [16]. DENV circulates globally as four serotypes: DENV-1, DENV-2, DENV-3, and DENV-4 [17]. Approximately 2 to 7 days after a mosquito bite, infection with any serotype of the virus can result in Dengue fever (DF), which may quickly progress to Dengue hemorrhagic fever (DHF), and if neglected or undetected, it can lead to Dengue shock syndrome (DSS) [18–20]. Dengue has evolved into a serious, potentially fatal consequence for people, as there is currently no vaccine that can effectively reduce the severity of the disease caused by all serotypes, and its prevalence has been steadily increasing [21].

Whenever the dengue virus infects the human host, both the innate (interferons, complement system, etc.) and adaptive (immunoglobulins and cytotoxic T-cells) immune systems are stimulated to neutralize the virus. In severe Dengue infection, the uncontrolled generation of inflammatory cytokines, leading to phenomenon termed ‘cytokine storm’, has been principally implicated as the causal agent of fatality. Antibodies produced against DENV play a crucial role in disease outcome, as during a secondary heterotypic infection (i.e., an earlier infection by one serotype followed by the current infection by another serotype of DENV), the virus causing the secondary infection cannot be efficiently neutralized by the antibodies against the first serotype. Furthermore, a phenomenon referred to as antibody-dependent enhancement, or ADE, may be enhanced by these non-neutralizing antibodies or low amounts of neutralizing antibodies. Several vaccines have been explored to treat dengue infection, including live attenuated tetravalent vaccines and Dengvaxia. However, developing a vaccine that is effective against all four dengue serotypes remains a challenge [22]. Importantly, vaccine efficacy varies from person to person, depending on age, serotype, and serostatus [23, 24]. Nevertheless, seronegative individuals were advised to avoid vaccination because it was reported that over time, the number of vaccine-induced antibodies decreased [25]. A potential ADE effect has been a significant restriction for the development of a prophylactic vaccine targeting DENV infection.

In spite of efforts by multiple research groups, there is still no therapeutic drug available in the market that can restrict virus replication or suppress the infection-induced ‘cytokine storm’ to reduce morbidity and/or mortality. Although various drugs, such as Chloroquine, Prednisolone, Balapiravir, Celgosivir, and Lovastatin have been tried, none of them showed promising results during clinical trials. Therefore, supportive medication is mainly used upon the diagnosis of Dengue infection. For example, treatment with paracetamol or pain medications can alleviate or reduce Dengue symptoms, such as muscle soreness, weariness, and fever. Ibuprofen and aspirin, two examples of non-steroidal anti-inflammatory medicines (NSAIDs), are commonly used, but they are not advised because they can exacerbate the prognosis of illnesses, resulting in adverse health conditions. By managing a patient’s fluid content, which is crucial for the management of severe DF, and providing proper medical care, symptoms and mortality can be reduced from more than 20% to less than 1% [1, 26]. Notwithstanding supportive therapy, DENV infection may result in endothelial dysfunction, antibody-dependent cellular cytotoxicity (ADCC), cytokine storm (hypercytokinemia), and irregular stimulation of the complement system (CS), leading to a more severe case of Dengue [27].

Addressing the various host-viral modifications at the molecular level may provide key insights into viral evolution, viral escape mechanisms, host-virus interactions, disease consequences, and public health readiness. This information may advance our understanding of viral infections and facilitate the development of efficient preventative, diagnostic, and therapeutic approaches. The current review will specifically focus on recent advances related to the modulation of epigenomic regulation, transcription, post-transcriptional regulation, as well as the translational and post-translational machinery of the host cell.

## DENV and the host epigenetic machinery

Epigenetic plays a vital role as it has the potential to regulate gene expression without altering the genetic sequences. Modulation of epigenetic regulation in the host-DENV interaction is noy yet completely understood. Recent years have seen growing evidence highlighting the pivotal role of epigenetic regulators in gene expression regulation. These regulators encompass various mechanisms such as DNA methylation, chromatin remodeling, histone modification, and regulatory RNA. A comprehensive understanding of the complex interactions among these epigenetic processes is essential for elucidating the consequences they exert on both host cells and DENV during infection. Such insights can aid in the identification of novel therapeutic targets.

**Fig. 3:**
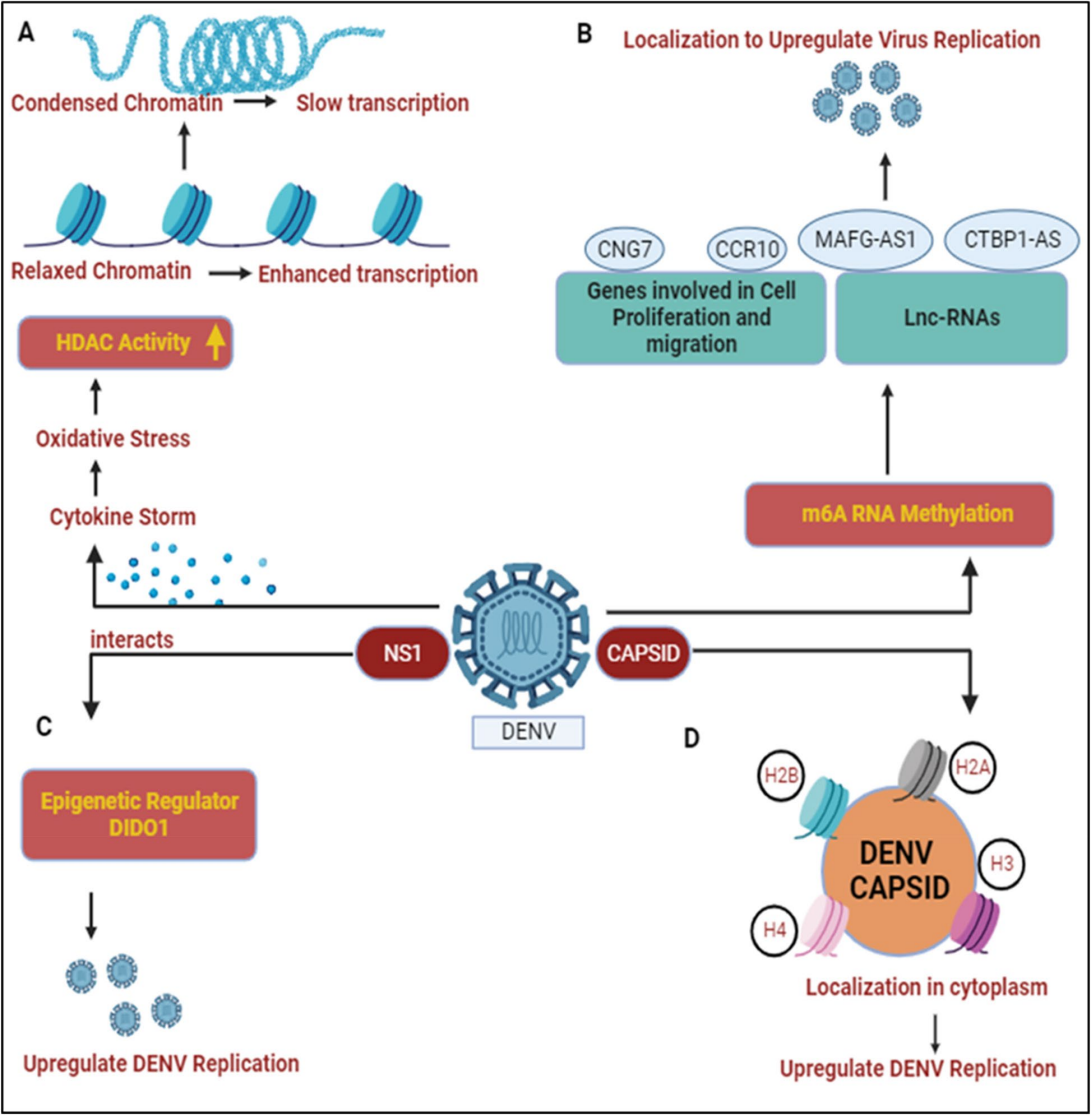
DENV and host epigenetic regulation: **A** DENV infection results in cytokine storm and leads to oxidative stress conditions in host cell, activating histone deacetylase (HDAC). HDAC activity condenses the chromatin and slows down the host transcription processes [28]. **B** DENV protein interacts with various host cellular genes/lnCRNAs and results in m6A RNA methylation. Their localization helps to enhance DENV replication [29, 30]. **C** DENV NS1 interacts with DIDO1, a master epigenetic regulator and somehow elevates DENV replication [35]. **D** Capsid protein interacts with core histones (H2A, H2B, H3, and H4) and localizes them in cytoplasm to participate in DENV replication and propagation [36]

Dinakaran et al. provided an overview that at the severe stage of infection, patients show symptoms characterized by inflammation and a cytokine storm resulting in oxidative stress, activating histone deacetylase (HDAC). HDAC factors such as DNA methyltransferase (DNMT) favor the silencing of promoters and slow down host gene transcription processes, as noted in Fig. 3A. This epigenetic modification is important for investigating mental health issues related to dengue infection. Various HDAC inhibitors like valproate, quetiapine, or carbamazepine are prescribed for treatment as HDAC/Dnmt inhibitors [28]. Comprehensive transcriptome analysis identified that DENV proteins interact with host genes (CCR10, CNG7, PLXNA3) and lncRNAs (CTBP1-AS, MAFG-AS1) engaged in various biological processes, such as the immune response, inflammation, and the cell cycle, as illustrated in Fig. 3B. The role of m6A methylation has been implicated in potentially regulating these genes/lncRNAs to enhance DENV replication, as noted in Table 1 [29, 30]. Other flaviviruses (YFV, WNV, and ZIKV) also contain m6A methylation, indicating a conserved mechanism within this virus family [31]. An epitranscriptome analysis showed that various kinds of methylation might be used to regulate flaviviral RNA [32]. It’s interesting to note that each flavivirus has a unique methylated nucleotide composition. For example, ZIKV vRNA does not include methylated uridines m^3^Um and m^5^Um, while DENV vRNA does [32]. On the other hand, dimethylcytosines m^5^Cm and m^4^_4_C are exclusive to ZIKV vRNA [32]. Since methylation plays an essential role in the Epstein-Barr virus (RBV) and the murine leukemia virus (MLV) [33, 34], it will be interesting to explore the possible roles of these alterations in DENV and ZIKV.

**Table 1:** Various molecular processes modulated by DENV proteins and host proteins to either enhance or hinder virus replication

**Molecular Processes**	**Viral proteins /UTRs**	**Host proteins /Components**	**Function**	**References**
**Epigenetics**		HDAC	Condense chromatin and slow down host transcription	[28]
		CNG7, CCR10, MARF-AS1, CTBP-AS	Localize in cytoplasm to enhance viral replication, proviral effect	[29, 30]
	NS1	DIDO1	NS1 and DIDO1 interaction upregulates viral replication	[35]
	Capsid	H2A, H2B, H3, H4	Capsid colocalize core histones to enhance viral replication	[36]
**Transcription**	Envelope	TAL-1	Hinder host transcription processes	[37]
		GATA-1, GATA-2, NF-E2	Affects the process of Megakaryopoiesis and decrease platelets production	[38]
		CDK8	Upregulates the glycolysis and metabolic enzyme production. Proviral effect	[39]
	DENV core protein	P-TEFb	Regulates DENV Pathogenesis	[40]
**Post-transcriptional**	NS5	snRNPs	Alters the process of splicing	[41]
	NS5	RBM10	Generate proviral isoforms via alternative splicing	[42]
	sfRNA	XRN1	Inhibit exonuclease activity and stabilize viral RNA	[43]
	NS3 and NS4A	IRAV and MOV10	Antiviral activity	[44]
**Translation**		eIF4E	Recognize 5’ Cap of viral RNA	[45]
	sfRNA	G3BP1, G3BP2, Caprin1	Inhibition of interferon stimulated gene (ISG) translation	[46]
	3’ UTR	PABP	Regulates translation of DENV RNA	[47]
**Post-translational**	NS3	TRIM69	TRIM69 targets NS3 for protesosomal degradation	[48]
	Envelope		Envelope glycosylation helps to attach with host receptors for viral entry, proviral effect	[49–51]
	NS1	C1s, C4, MBL	Glycosylated NS1 interacts with C1s, C4, MBL inhibits interferon related response, showing proviral effect	[52]
	NS5	STAT2	Target proteosomal degradation, antiviral effect	[53]
		STAT3	Negatively regulate Type I IFN and Type III interferon responses and enhance viral propagation	[54]
**Stress Responses**	NS3	TIA1, TIAR	Colocalize to enhance viral propagation	[55]
	3’UTR	G3BP1, G3BP2, DDX6, Caprin1, USP10	Colocalize to enhance viral propagation	[56]
	NS4B	VCP1-NPL4	Proviral effect	[57]
	NS1	ATF3	Contributes to neuroinflammation during dengue infection	[58]

Dengue non-structural genes participate in enhancing virus replication by modulating various epigenetic targets. Localization studies have shown that NS1 binds with the epigenetic regulator DIDO1 (Death Inducer Obligator 1) to promote virus replication, as shown in Fig. 3C. The silencing of DIDO1 results in reduced virus replication, indicating its significant role [35]. The dengue structural protein capsid is found to localize in the nucleus and target the four core histones, H2A, H2B, H3, and H4. DENV capsid forms heterodimers with histones and disrupts nucleosome formation for its own amplification, as shown in Fig. 3D, Table 1 [36]. Gomes et al. provided an overview that dengue-infected patients with symptoms of haemorrhagic manifestations showed demethylation of the TNFα promoter gene and IFN-γ, leading to acute inflammation resulting in the production of a cytokine storm during dengue infection [59, 60].

## DENV and the host transcriptional machinery

**Fig. 4:**
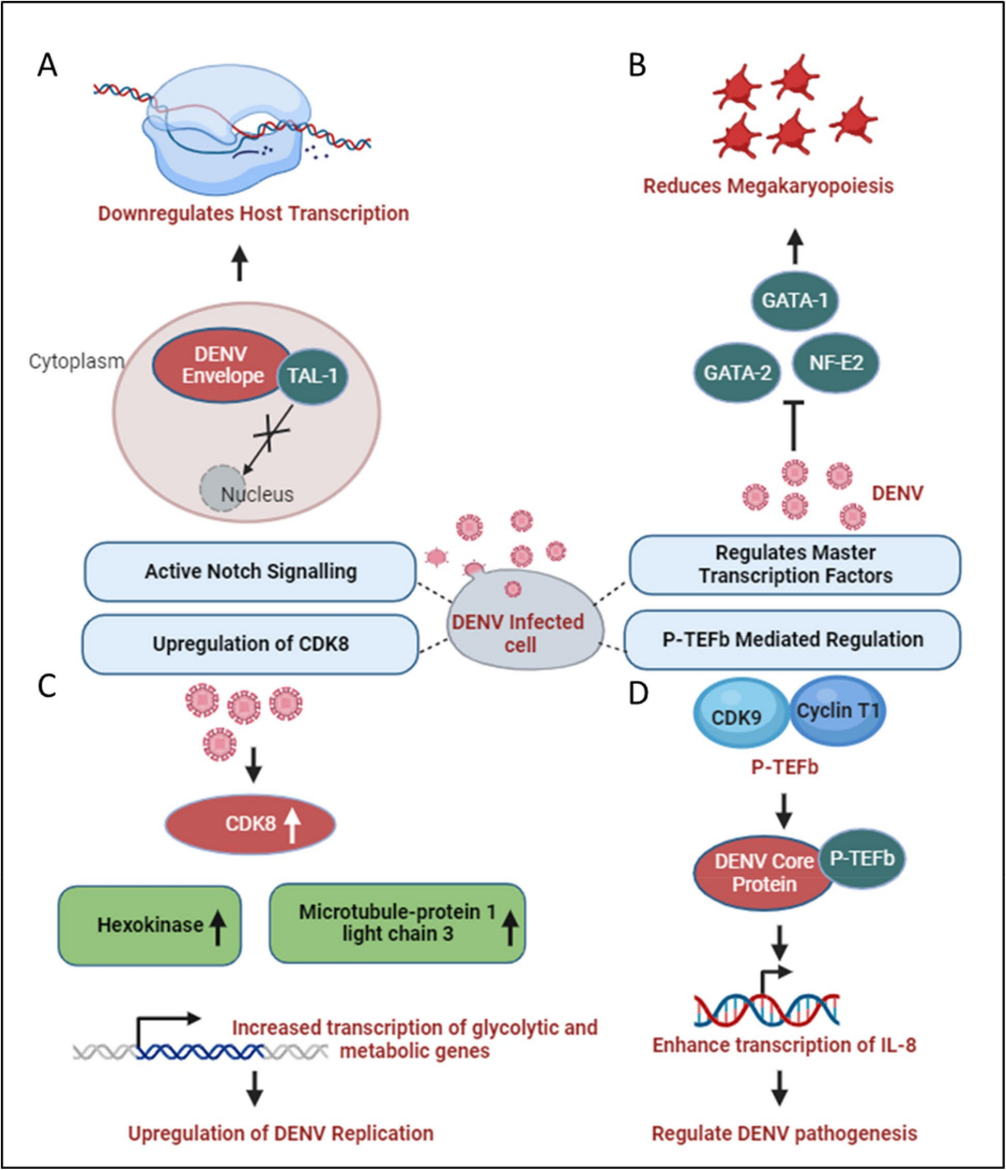
DENV and host transcriptional regulation: **A** DENV Envelope sequesters the transcription factor TAL-1 in the cytoplasm, affecting its transcriptional function in the nucleus [37]. **B** Various factors involved in transcription such as GATA-1, GATA-2, and NF-E2, which participate in the process of megakaryopoiesis, are dysregulated during dengue infection [38]. **C** During DENV infection, glucose and metabolic processes increase, enhancing RNA Polymerase II activity. This leads to higher expression of genes like Hexokinase and Microtubule-associated protein 1 light chain 3, promoting increased transcription of metabolic genes and enhancing DENV replication [39]. **D** P-TEFb interacts with DENV and activates NF-kB elements within the promoter region of IL-8 gene, enhancing IL-8 transcription which may play a significant role in DENV pathogenesis [66]

Human pathogenic viruses have evolved various mechanisms to hijack host cell gene expression at the transcriptional level to facilitate their life cycle. Although the DENV genomic RNA or viral replication complexes are localized exclusively to ER-associated regions of the cytosol, structural and non-structural proteins encoded by the virus migrate to the nucleus and interact with different host proteins. Among DENV proteins, at least two, namely the C and NS5, distribute both in the cytoplasm and nucleus, getting involved in protein-protein interactions that cause the regulation of gene expression [61]. The C-protein is enriched in the nucleolus, interacting with the host protein nucleolin and facilitating viral morphogenesis [62]. Additionally, the C-protein has been indicated to mimic histone proteins and interfere with nucleosome formation, thereby influencing host cell transcription [36]. During virus infection, chemokine levels are elevated as an immune response against viruses. For instance, Interleukin-8 (IL-8) levels are upregulated in patients following infection by different group of RNA viruses such as Coronaviruses and Cytomegaloviruses to promote virus replication [63, 64]. Similarly, In DENV-infected patients, higher IL-8 levels have been shown to correlate with severe DHF [65]. Li and co-workers showed a role for the C-protein in augmenting IL-8 transcription by associating with Positive Transcription Elongation Factor-b (P-TEFb), a complex of CDK9 and Cyclin T1 that promotes gene expression through augmentation of RNA Pol-II processivity [66]. Since pharmacological inhibition of P-TEFb showed a reversal of IL-8 upregulation, this can be a potential therapeutic strategy to avert the emergence of severe DENV, as illustrated in Fig. 4D [40, 66]. Although the significance of nucleolar enrichment is not clear yet, it is possible that, as observed in other RNA viruses, the DENV C-protein influences host ribosome assembly, prioritizing translation of the viral positive-strand RNA over host mRNAs [67]. Although there is no evidence that the DENV E-protein migrates to the nucleus, it has been suggested to influence host cell transcription through an interaction with TAL-1, a transcription factor involved in megakaryopoiesis, as shown in Fig. 4A. In thrombocytopenia, only naive MEG-01 cells could be infected by DENV, and differentiated cells were resistant to virus infection/replication and it slowed the progress of megakaryopoiesis [37]. It has been observed that DENV infection enhances the activity of the Notch signaling pathway in the human system, and has been shown to limit the process of megakaryopoiesis. As, Notch-1 is known to regulate not only its downstream component RBPjk33, but also the megakaryopoiesis-specific transcription factor TAL-1. The sequestering of TAL-1 in the cytoplasm by DENV E protein may affect its availability for its transcription-related function in the nucleus, thus impeding the megakaryopoiesis. Dengue Virus also dysregulates master transcription factors involved in platelets development and maturation such as NF-E2, GATA-1, and GATA-2 and the PI3K/AKT/mTOR signaling pathway as depicted in Fig. 4B [38].

DENV exhibits distinct characteristics among its four serotypes, with DENV2, in particular, showcasing a reliance on increased glucose utilization and enhanced glucose metabolism to produce the necessary metabolic intermediates essential for viral replication [68]. During DENV-2 infection, there is an elevation in the expression of specific metabolic enzyme-encoding mRNAs, especially glucose-6-phosphate and fructose-6-phosphate in infected cells, through an upregulation in the expression of glucose transporter GLUT1 (which transports glucose from the extracellular environment into the cytoplasm) and hexokinase 2, the first rate-limiting enzyme in the glycolytic pathway [69]. DENV replication requires high glucose and metabolic intermediate for transactivation of RNA polymerase II [39]. Butler and co-workers confirmed the transcriptional upregulation of HK2 following DENV infection in a different model system and additionally showed an upregulation of Cyclin-dependent kinase 8 (CDK8) levels in DENV-infected cells to be upstream of HK2 [39]. Furthermore, the upregulation of CDK8 was shown to be beneficial for virus replication since a reversal led to a reduction in viral titers. As CDK8 expression increases, in addition to that of HK2, there is a concurrent upregulation of crucial genes like microtubule-associated protein 1 light chain 3 (LC3) and other proteins essential for virus replication. This coordinated effect is illustrated in Fig. 4C [39].

In a study conducted by A. Carlin, the focus was on interferon-regulatory factors (IRFs), a family of transcription factors known for their role in generating type I interferon (IFN) and eliciting antiviral responses. The investigation involved triple knockout (TKO) mice, genetically engineered to lack three key transcription factors that regulate type I IFN production. Surprisingly, the TKO mice, deficient in the factors controlling type I IFN production, demonstrated increased survival rates when challenged with dengue virus (DENV). This finding strongly suggests that the antiviral effects of IRFs are mediated through interferon responses during DENV infection [70]. Contrastingly, the study also revealed a strategic countermeasure employed by the dengue virus to facilitate its replication and propagation. Dengue virus actively suppresses interferon-stimulated genes by impeding the recruitment of the transcription factor PAF1C. Additionally, the virus modulates SEC61, a key cellular component, leading to the inhibition of DENV replication. This dual nature of the host-virus interaction underscores the intricate balance between the host’s antiviral defenses, mediated by IRFs and interferon responses, and the virus’s ability to subvert these defenses for its replication advantage [61].

Depending on the model system used, DENV infection is reported to either augment or decrease the transcriptional activity of NFE2L2, encoded by the NRF2 gene. In monocyte-derived dendritic cells (moDCs), it inhibits NFE2L2 activity, whereas in differentiating megakaryocytes, it seems to augment NFE2L2 activity [71, 72]. This manipulation is particularly prominent during instances of dengue fever (DF) and dengue hemorrhagic fever (DHF). DF is characterized by thrombocytopenia, a condition marked by a low platelet count, primarily resulting from the process of megakaryopoiesis. Despite lacking a nucleus, platelets contain essential cellular components such as cytosolic ribosomes, endoplasmic reticulum (ER), Golgi apparatus, and mitochondria, the cell’s powerhouse. These components provide the necessary machinery for viral replication and transcription [73]. In individuals infected with the dengue virus (DENV), platelets serve as a reservoir for the infectious DENV antigen. However, this aspect is controversial because other studies could not corroborate it. Consequently, severe thrombocytopenia is a prominent symptom of dengue with potential complications leading to dengue shock syndrome (DSS) in certain cases.

## DENV and the host post-transcriptional machinery

Modulation of post-transcriptional gene regulation mechanisms within host cells, including alternative splicing, mRNA transport, mRNA degradation, and RNA silencing, plays a crucial role in the Dengue virus (DENV) life cycle. An understanding of these mechanisms is key to unraveling the complex interplay between the virus and its host. Among the non-structural proteins encoded by the DENV genome, NS5 and NS3 have been reported to influence host cell post-transcriptional machinery, facilitating virus replication.

**Fig. 5:**
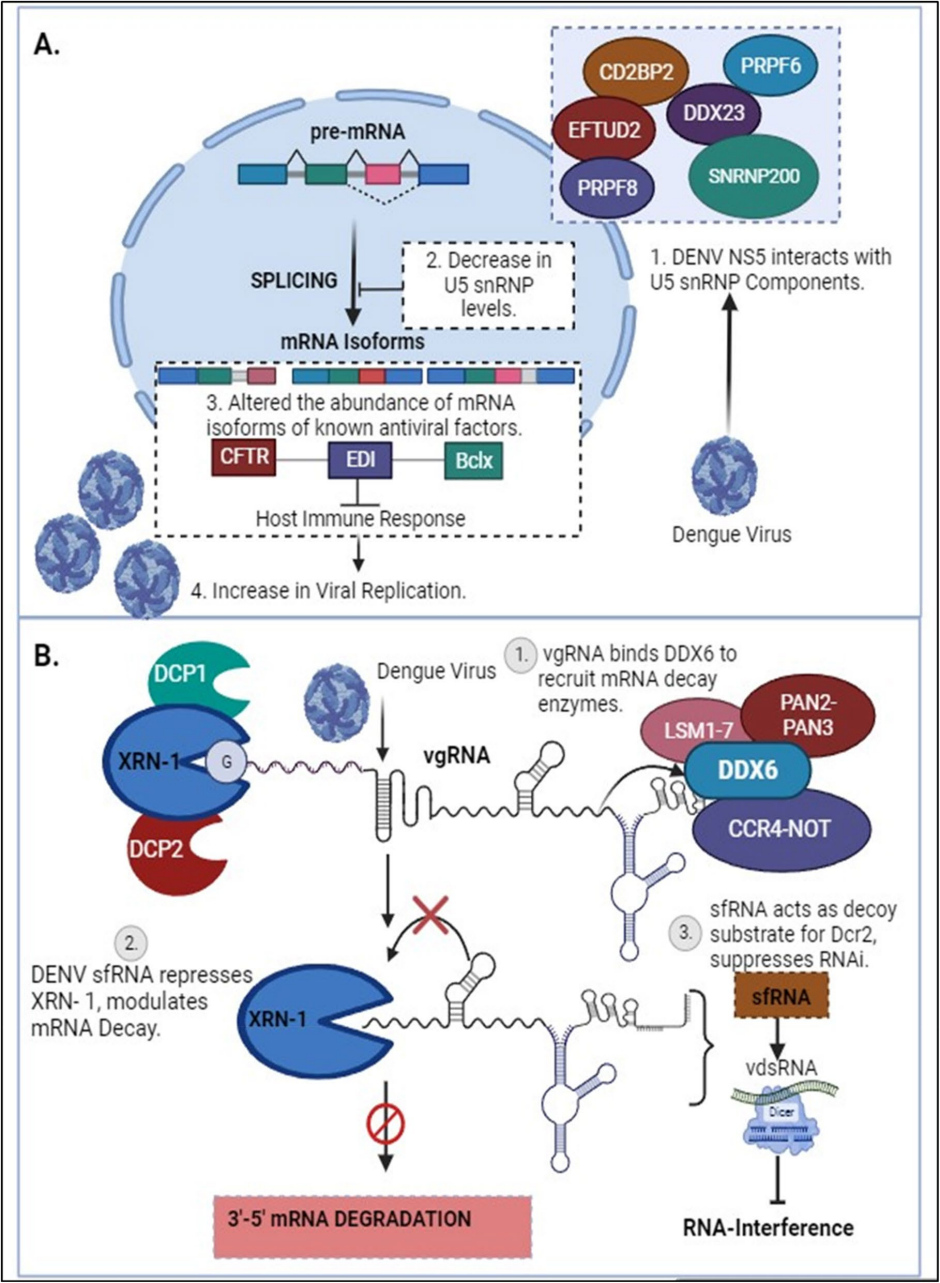
DENV and host post-transcriptional regulations: **A** Dengue Virus NS5 associates with active spliceosomes, interacting with key components of the U5 snRNP and sequestering them in the cytoplasm. This reduces their levels in the nucleus and alters the events of alternative splicing [41]. **B** In the early stages of DENV infection, viral genomic RNA (vgRNA) binds to DDX6 via the 3’UTR and recruits mRNA decay enzymes to the viral replication complex. The exoribonuclease XRN-I initiates the degradation of vgRNA, resulting in the formation of sfRNA. These pseudoknot structures play a role in stalling the XRN1 enzyme near the 5’ border of the 3’ UTR, causing the inhibition of XRN-1 and leading to modulations in mRNA degradation pathways, consequently affecting the RNAi response [43]

In addition to its unique role in replicating viral genomic RNA (both negative and positive-sense strands) in ER-associated Replication Complexes (RC) and selectively capping positive-sense genomic RNA, NS5 localizes to the host nucleus with the aid of two Nuclear Localization Signals (NLS). This localization influences the splicing of select host cell pre-mRNAs [41]. Pre-mRNA splicing stitches gene exons together to generate unique mRNAs and is catalyzed by large RNA-protein spliceosomal complexes, termed either major (U2-dependent) or minor (U12-dependent) spliceosomes. The major spliceosome is responsible for removing 99.5% of the introns [74]. Splicing also affects mRNA transport to the cytoplasm and decoding by the translation machinery. The NS5 interactome includes core components of U5 small nuclear ribonucleoprotein (U5snRNP) complex such as CD2BP2, DDX23, etc. [41], Fig. 5A. This modulates alternative splicing of known host antiviral factors like Cystic Fibrosis Transmembrane Regulator (CFTR), EDI (extradomain A of fibronectin), and Bclx (B-cell lymphoma-extra-large), potentially impacting the host’s immune response to Dengue infection [41]. By modulating the activities of host splicing factors, DENV can orchestrate specific splicing patterns that enhance viral gene expression and protein production [75–77].

Polyamines (spermidine/spermine) are ubiquitous small, positively charged molecules that can function as chaperones for cellular nucleic acids [78, 79]. Multiple viruses are known to depend on this function of polyamines for genome replication [80]. The enzyme Spermidine/spermine-N1-acetyltransferase (SAT1), an Interferon-sensitive gene (ISG), acetylates polyamines, leading to a drop in intracellular levels and thereby inhibiting viral genome replication [81, 82]. However, SAT1 expression is downregulated upon the inclusion of exon-4 of the gene in mature mRNA, an incorporation prevented by the splicing factor RBM10 [81]. As a counter to SAT1-mediated reduction in intracellular polyamine levels, DENV NS5 interacts with RBM10, mediating its degradation by the proteasome, leading to an increase in the abundance of SAT1 mRNA containing exon-4 and a consequential reduction in SAT1 protein levels [42]. Additionally, NS5 affects the splicing efficiency of RIG-I, a sensor of viral RNA in the cytoplasm, which functions in inducing an antiviral immune response [41]. In this context, it is intriguing to note that NS5 reduces the splicing efficiency of endogenous RIG-I mRNA and also increases the expression of dominant-negative forms of IKKε (inhibitor of nuclear factor kappa-B kinase ε) during DENV infection. This, by all means, results in supporting pro-viral conditions in the cell. Hence, it can be appropriately proposed that the interaction of NS5 with the splicing machinery is an immune suppression strategy [41]. Extending these studies to include other members of the flavivirus family, a study by Michalski et al. suggested that ZIKV sfRNAs also affect host mRNA splicing. ZIKV sfRNAs can sequester splicing factors like PHAX, PPIH, SF3B1/2, and NMP1, which are involved in regulating alternative splicing of host transcripts, significantly contributing to the mis-regulation of cellular RNA splicing, including exon-skipping and intron retention events in various genes during infection [83].

In response to virus infection, host cells secrete interferons, which bind to cognate receptors on the cell surface and activate the expression of IFN-sensitive genes through signal transduction. STAT2 forms an essential component of this signal transduction pathway, and suppression of this protein leads to a suppression of the innate host antiviral response. While in the cytosol, NS5 associates with the host STAT2 protein, catalyzing its degradation and the consequent silencing of STAT2-dependent gene expression, in the nucleus, NS5 interacts with a multiprotein complex involved in STAT2-independent gene expression and negatively regulates its function [53]. Petit and coworkers showed that through interaction with PAF1, a component of the Polymerase Associated Factor 1 complex (PAF1C), which is instrumental in the expression of STAT2-independent immune response genes, DENV NS5 inhibits its function, leading to suppression of the antiviral genes targeted by PAF1C [84].

During dengue infection, the virus interacts with the host cellular machinery responsible for 5’ capping. DENV employs multiple strategies to hijack and manipulate this process. For instance, the virus sequesters essential host factors involved in capping, such as the cap-binding complex and associated enzymes. By doing so, DENV ensures efficient capping of its own viral transcripts, enhancing their stability and translational competence [85]. During dengue infection, the antibodies produced are generally cross-reactive among DENV serotypes, carrying a higher risk of promoting Antibody-Dependent Enhancement (ADE) [86]. A study by Narayan and Tripathi, demonstrated that dengue antibody-dependent enhancement (ADE)-mediated virus entry predominantly causes the enrichment of differentially expressed genes (DEGs) that regulate RNA processing. Many of these factors are known to interact with dengue viral RNA, suggesting that dengue ADE modulates the transcriptome of monocytes to upregulate genes responsible for vesicular transport and mRNA processing [87]. Additionally, the spatial regulation of mRNA transport during dengue infection is mediated by the interaction between viral proteins and host factors involved in mRNA transport processes. For example, RNA-binding proteins (RBPs) are key regulators of mRNA transport and localization. HnRNPs (heterogeneous nuclear ribonucleoproteins), for instance, have been shown to regulate host mRNA expression in response to dengue infection. Modulation of these RBPs by dengue infection can impact mRNA transport and localization, potentially influencing viral replication and pathogenesis. Furthermore, mRNA transport in Dengue infection can influence the host immune response. Viral RNA molecules transported to specific subcellular compartments may interact with immune sensors, such as pattern recognition receptors (PRRs), triggering antiviral immune responses. In addition, the cytosolic localization of viral mRNA molecules can impact the accessibility of viral RNA to host immune factors, influencing the recognition and clearance of the virus [88].

One pivotal facet of post-transcriptional regulation is mRNA turnover, which involves the mRNA decay machinery to synchronize gene expression by balancing the stability and lifespan of mRNAs through regulated mechanisms. Efficiently evolving viruses exploit the cellular gene expression machinery to escape antiviral responses and control viral replication [89]. Viral genomic material, in addition to virus-encoded factors and enzymes, interacts with elements of the host mRNA decay pathways to modulate the half-life of both viral and cellular mRNAs. Furthermore, viruses encode ribonucleases to degrade cellular mRNAs, providing multiple advantages, one of which is increased stability of viral transcripts. Additionally, it has been demonstrated that the dengue virus regulates XRN1 (5’-3’ exoribonuclease 1 protein), a key component of the cellular mRNA decay machinery responsible for degrading mRNAs in the 5’ to 3’ direction. DENV undertakes this modulation through subgenomic flavivirus RNA (sfRNA), a non-coding RNA molecule derived from the 3’ untranslated region (UTR) of the viral genome. This sfRNA can bind directly to XRN1, inhibiting its exonuclease activity. By inhibiting XRN1, sfRNA prevents the degradation of viral RNA, including non-coding regions, allowing the virus to maintain a stable pool of genomic RNA for replication. Additionally, by inhibiting XRN1, the virus stabilizes host mRNA, hindering the process of mRNA degradation and potentially dampening the host immune response, as shown in Fig. 5B [43]. This particular mechanism of stalling and repression of XRN1 by generating sfRNA appears to be a common strategy by many members of Flavivirus family. Zika virus targets the cytoplasmic 5’-3’-exoribonuclease XRN1, by interacting with the cytoplasmic RNA decay machinery, which results in the suppression of gene expression and the generation of copious amounts of sfRNA in infected cells. Notably, XRN1 targeting is not limited to the Flaviviruses but is employed by RNA viruses from diverse evolutionary backgrounds as part of the molecular arms race to help the infecting virus usurp the innate immune response of the host. Further studies, elucidate that DENV-2 sfRNAs interact with and sequester two viral restriction factors involved in aspects of cellular RNA decay, DDX6 and EDC3, in addition to a deadenylase component CNOT1. Similar results have been obtained with the ZIKIV sfRNA, demonstrating that besides the RNA decay pathway, these viruses also reduce the decapping and deadenylation of transcripts [83].

During dengue infection, several mRNA degradation pathways have been implicated, including exonucleolytic degradation mediated by the exosome complex, endonucleolytic cleavage by endoribonucleases, and the miRNA-mediated decay pathway [90]. To aid viruses with their establishment inside the host, these viruses encode several proteins that interact with various host factors. One such class of proteins identified as Viral Suppressors of RNA Silencing (VSR) has been demonstrated to interact with components of the host machinery participating in post-transcriptional gene silencing, a known antiviral defense mechanism. Based on the present study, it has been shown that dengue NS3, a known VSR, interacts with a cellular chaperone, Heat shock protein family A (Hsp70) member 1A (HSPA1A), subsequently modulating its expression levels as well [91]. Moreover, it was discovered that HSPA1A is linked with the host RNA silencing machinery by coordinating with the Argonaut proteins, Ago1, and Ago2, and translocating to the cytoplasm of the cell along with them. Hence, these outcomes provide evidence for the participation of other host partners in mediating the VSR role of dengue NS3 and in determining the mechanisms of RNA silencing in the case of Dengue virus infection. Furthermore, one of the key mediators of antiviral immunity is Interferon-stimulated genes (ISGs) that play a crucial role in the immune response to DENV infection. IRAV is an RNA-binding protein, which functions as an ISG and localizes to cytoplasmic processing bodies (P bodies) in uninfected cells, where it interacts with the MOV10-RISC complex RNA helicase (MOV10) and Upstream frameshift 1 (UPF1), a key component of nonsense-mediated RNA decay pathways. IRAV and MOV10 are believed to function in conjunction with other proteins like HuR, LARP1, and PABPC (some RNA-binding proteins involved in the mRNA decay pathway) to operate in the disruption of viral RNA by colocalizing with the DENV replicating complex, particularly NS3 and NS4A, thus possessing anti-viral activity. A study by Balinsky et al. in 2017 proposed that IRAV is upregulated during DENV infection [44].

## DENV and the host translational machinery

The Dengue virus, functioning as an intracellular parasite, has developed sophisticated strategies to manipulate the host’s translational machinery, ensuring efficient viral protein synthesis and replication. This intricate interplay between the virus and the host’s translation machinery is crucial for the successful propagation of the virus within the host cell. Upon infection, the Dengue virus genome, a positive-sense single-stranded RNA, serves as a template for the translation of viral proteins. Translation initiation primarily occurs through the cap-dependent translation mechanism [92]. In this process, the cap-binding protein complex (CBC), specifically the eIF4E factor, recognizes the cap structure at the 5’ end of the viral RNA. Subsequently, the eIF4F complex, comprising eIF4E, the adapter protein eIF4G, and the helicase eIF4A (along with its cofactor eIF4B), binds to the cap and recruits ribosomes to the viral mRNA, facilitating translation initiation [45, 93].

In addition to the aforementioned mechanisms, Bidet and Garcia-Blanco identified the DENV-2 NS3 protein as an interacting partner of various translation initiation factors, namely eIF4G, eIF5A, and eIF3L, showcasing the importance of these proteins in upregulating viral RNA translation. This observation underscores DENV-2’s ability to target key components of the host’s translation initiation machinery [94]. Furthermore, these findings extend beyond DENV; the NS5 protein of the Yellow Fever virus (YFV) was also found to interact with these translation factors in two-hybrid screening assays [95]. Translation elongation factors, such as eEF1A and eEF2, have also been shown to be essential for WNV and Yellow Fever Virus 17D (YFV17D), respectively, confirming the importance of these protein categories in flaviviral infections, besides the capacity of flaviviruses to manipulate the host’s translational apparatus [96].

The availability of eIF4E is a key control point for translation. Hypophosphorylation of eIF4E-binding proteins (4E-BPs) prevents their interaction with eIF4E in the cap-binding complex, resulting in the suppression of cap-dependent translation. In such situations, an internal ribosome entry site (IRES) is utilized to initiate cap-independent translation. IRES-mediated translation involves unique sequences present in the Dengue virus 5’-untranslated region (UTR), which recruit ribosomes directly [97, 98]. A proposed non-canonical model suggests that during low levels of eIF4E, RNA sequences in the 3’ UTR stabilize translation initiation factors at the 5’ end, subsequently recruiting eIF4G and eIF4A, thereby bypassing the requirement for eIF4E. This process facilitates the formation of a bridge between the 5’ UTR and the 3’ UTR, enhancing translation under these conditions [98]. A similar phenomenon is observed in other genera within the Flaviviridae family; hepatitis C virus (HCV) contains an IRES to facilitate translation initiation. HCV recognizes the 43S particle primarily through IRES RNA interaction. This direct interaction helps in initiating translation processes [99]. Interestingly, Polypyrimidine tract-binding protein (PTBP) protein helps other viruses, such coxsackievirus B3 (CVB3), circularise the viral RNA by bridging the UTRs necessary for effective translation of viral RNA [100].

The Dengue virus 3’ UTR stimulates translation through both cap-dependent and cap-independent mechanisms. Furthermore, the 3’ stem-loop structure complements this process by enhancing polysome formation. While the lack of a 3’ poly(A) tail in the Dengue virus genome is not entirely clear, the 3’ stem-loop domain is functionally analogous to a 3’ poly(A) tail, as it recruits translation initiation factors [97]. Notably, the 3’ stem-loop functions at both the translation and replication levels, as the Dengue virus RNA serves as both mRNA and a template for negative-strand synthesis [101]. In contrast CVB3 contains hexa-nucleotide stretch within stem loop C, which is critical for CVB3 IRES mediated translation [102]. Multifunctional RNA-binding proteins (RBPs) such as G3BP1 (G3BP Stress Granule Assembly Factor 1), G3BP2 (G3BP Stress Granule Assembly Factor 2), and Caprin1 are identified as novel regulators during Dengue virus type 2 (DENV-2) infection. They play a significant role in interferon-stimulated genes (ISGs) translation. It has been reported that DENV-2 sub-genomic flaviviral RNA binds to G3BP1, G3BP2, and Caprin1, leading to the inhibition of ISG mRNA translation, resulting in a proviral effect [46].

A notable phenomenon observed in Dengue virus translation is the binding of the poly (A)-binding protein (PABP) to the non-polyadenylated 3’ untranslated region (3’ UTR) of the viral RNA. Specifically, PABP interacts internally with the 3’ UTR, positioning itself upstream of the conserved 3’ stem-loop structure near the two dumbbell structures. The PABP-interacting protein 2 (PAIP2), a translation inhibitor specific to PABP, plays a role in interfering with the interaction between the Dengue virus 3’ UTR and PABP. *In-vitro* experiments using baby hamster kidney cell extracts revealed that PAIP2 inhibits the translation of Dengue virus reporter RNAs in a dose-dependent manner, underscoring its impact on the translational process. These findings shed light on the broader understanding of PABP’s translation mechanism, demonstrating its ability to bind to viral RNA even in the absence of a terminal poly(A) tail. This insight illuminates the intricate regulatory mechanisms governing the translation of Dengue virus RNA and provides potential avenues for therapeutic interventions [47].

## DENV and the host post-translational machinery

**Fig. 6:**
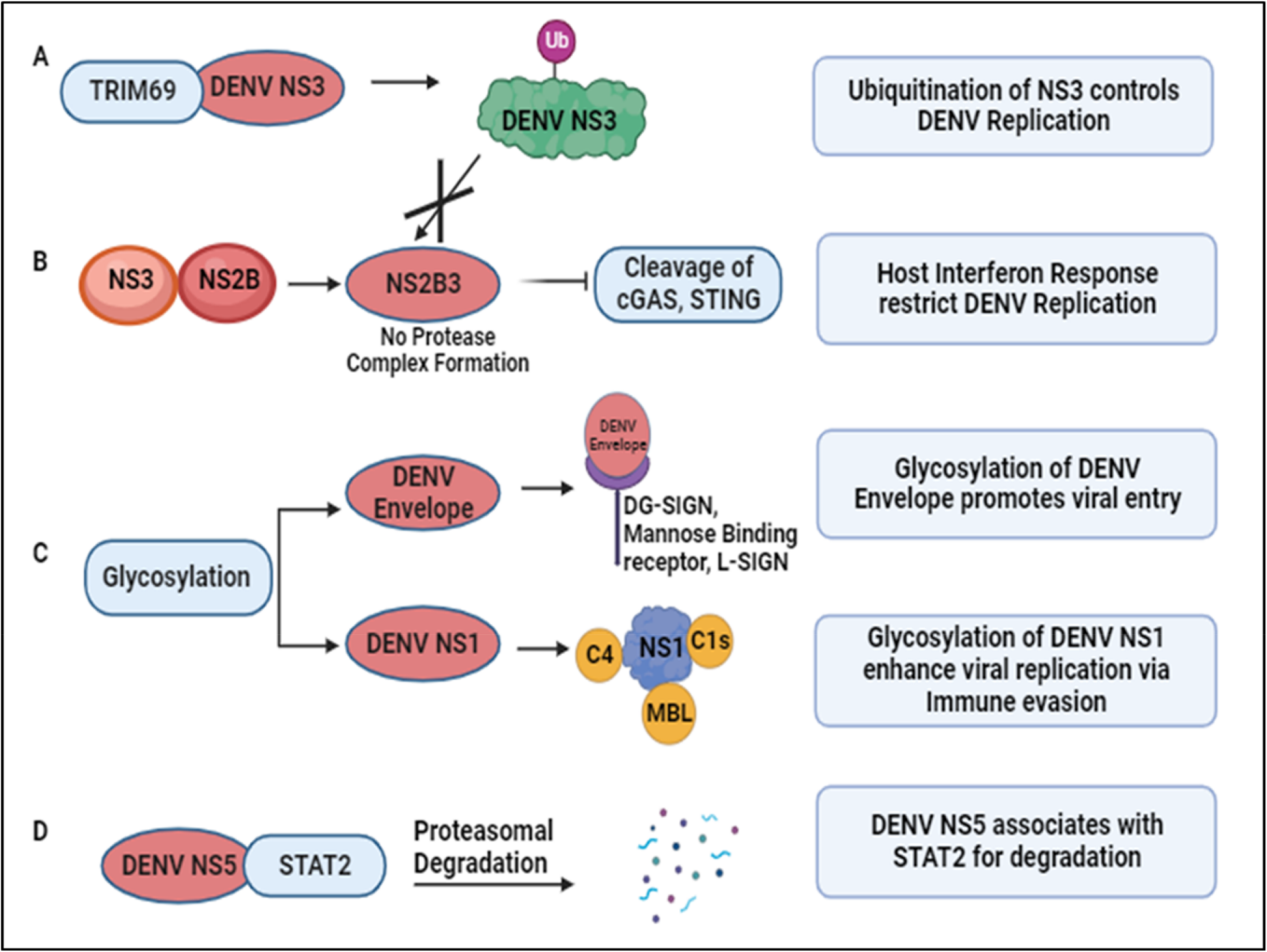
DENV and host post-translational regulation: **A** DENV NS3 interacts with TRIM69, induced as an Interferon Stimulated Gene (ISG), activating the ubiquitination pathway to restrict DENV replication [48]. **B** Conversely, ubiquitination of NS3 prevents the formation of the protease complex (NS2B3), inhibiting the cleavage of Interferon response-related genes, namely cGAS and STING. This results in an elevated host interferon response, targeting DENV replication [104, 105]. **C** Glycosylation of DENV proteins aids attachment to host surface receptors and various complementary proteins, contributing to immune evasion and viral propagation [49–52]. **D** DENV NS5 interacts with STAT2, preventing its phosphorylation and targeting it for proteasomal degradation [53, 106]

The genome of the Dengue virus initiates the formation of a single polypeptide through translation, subsequently processed to yield various functional and non-functional proteins. These proteins undergo post-translational modifications (PTMs), as depicted in Fig. 6. Given DENV’s lack of its enzymatic machinery for PTMs, it relies on the host’s PTM system for survival [103]. These modifications involve the covalent addition of small protein molecules or functional groups to specific amino acid residues, including ubiquitination (addition of ubiquitin), lipidification (addition of lipid), glycosylation (addition of carbohydrate), and chemical group modifications such as hydroxylation, phosphorylation, acetylation, and methylation [49].

During infection, these modifications can exert either proviral or antiviral effects by activating or inhibiting proteins or modulating the immune response. An example is the TRIM family protein TRIM69, an interferon-stimulated gene (ISG). TRIM family members, pivotal in the innate immune pathway, play diverse roles from transcription to post-translation. During DENV infection, TRIM69 interacts with DENV NS3, activating the ubiquitination pathway and restricting DENV replication, as illustrated in Fig. 6A [48]. Ubiquitination involves covalently attaching ubiquitin to the C-terminal glycine residue of the target protein. DENV NS3, in association with NS2B, forms a protease complex (NS2B3) that cleaves cyclic GMP-AMP synthase (cGAS) and Stimulator of Interferon Genes (STING). Ubiquitination of DENV NS3 prevents its proteolytic degradation, enhancing viral replication. However, reduced degradation of STING and cGAS enhances the interferon response, acting as host defense mechanisms against DENV as noted in Fig. 6B [104, 105]. To target the host cell, DENV utilizes the host’s glycosylation machinery for glycan conjugation on its proteins. Glycosylation, involving attaching glycan moieties to serine or threonine amino acid residues, plays critical roles in DENV entry, assembly, virulence, and pathogenicity. Receptors such as DC-SIGN, mannose-binding receptors, and L-SIGN receptors facilitate DENV entry by recognizing glycans on envelope proteins as shown in Fig. 6C [49–51]. Glycosylated DENV NS1 contributes to protein secretion and stability, binding to complement components and preventing lectin complement activation and DENV neutralization, regulating DENV pathogenesis [52].

Studies reveal novel mechanisms of immune evasion and potential antiviral therapeutic targets. DENV NS5 associates with Signal Transducer and Activator of Transcription 2 (STAT2), a critical component in interferon signaling, preventing its phosphorylation and targeting it for proteasomal degradation, as depicted in Fig. 6D [53, 106]. Additionally, STAT3 upregulation and activation negatively regulate Type I and Type III interferon responses during DENV-2 infection, suggesting STAT3 as a proviral factor for DENV propagation and a potential antiviral target [54]. Through immunoprecipitation coupled with mass spectrometry analysis, Zhang et al. identified host proteins exhibiting differential ubiquitylation patterns in DENV-infected cells. Notably, they found that the lipid droplet-localized type-III membrane protein AUP1 undergoes deubiquitylation upon DENV infection. This virus-induced deubiquitylation leads to increased AUP1 levels and subsequent accumulation within autophagosomes. Importantly, AUP1 interacts with DENV NS4A protein, promoting viral production. Interestingly, a ubiquitin-modified mutant of AUP1 suppressed the interaction with NS4A, resulting in impaired lipophagy and defective viral replication. These findings implicate ubiquitination as a critical host regulatory mechanism that DENV modulates to facilitate its replication cycle. Further studies are needed to fully elucidate the ubiquitylation changes induced by flaviviral infection [107].

In a study employing MS-based phosphoproteomics, the global host phosphorylation profile was investigated for DENV [108], WNV [109], and ZIKV, revealing shared cellular pathways whose phosphorylation status alters across different flaviviral infections, affecting various RNA processing and metabolic pathways [110]. Using the same strategy, the authors identified 604 host proteins differentially phosphorylated in JEV infected human U251 glial cells. Importantly, pharmacological inhibition of the JNK1 pathway in mice infected with JEV resulted in reduced inflammatory cytokine secretion, lower viral load, and increased survival rates. This indicates that the JNK1 signaling pathway, modulated by JEV-induced phosphorylation events, plays a critical role in driving the inflammatory response and pathogenesis during JEV infection [108].

## DENV and the host stress responses

DENV relies on host cellular processes for both its replication and translation. Upon infection, DENV intricately interacts with the host’s immune system, triggering a cascade of cellular and molecular responses aimed at combating the invading pathogen. Among these responses, host stress responses play a crucial role in modulating the outcome of DENV infection. Stress Granule (SG) formation is one of the consequences of cellular stress, heat shock, dysregulation of molecular mechanisms, and certain viral infections. Alterations in protein expression mechanisms results in SG assembly [111].

**Fig. 7:**
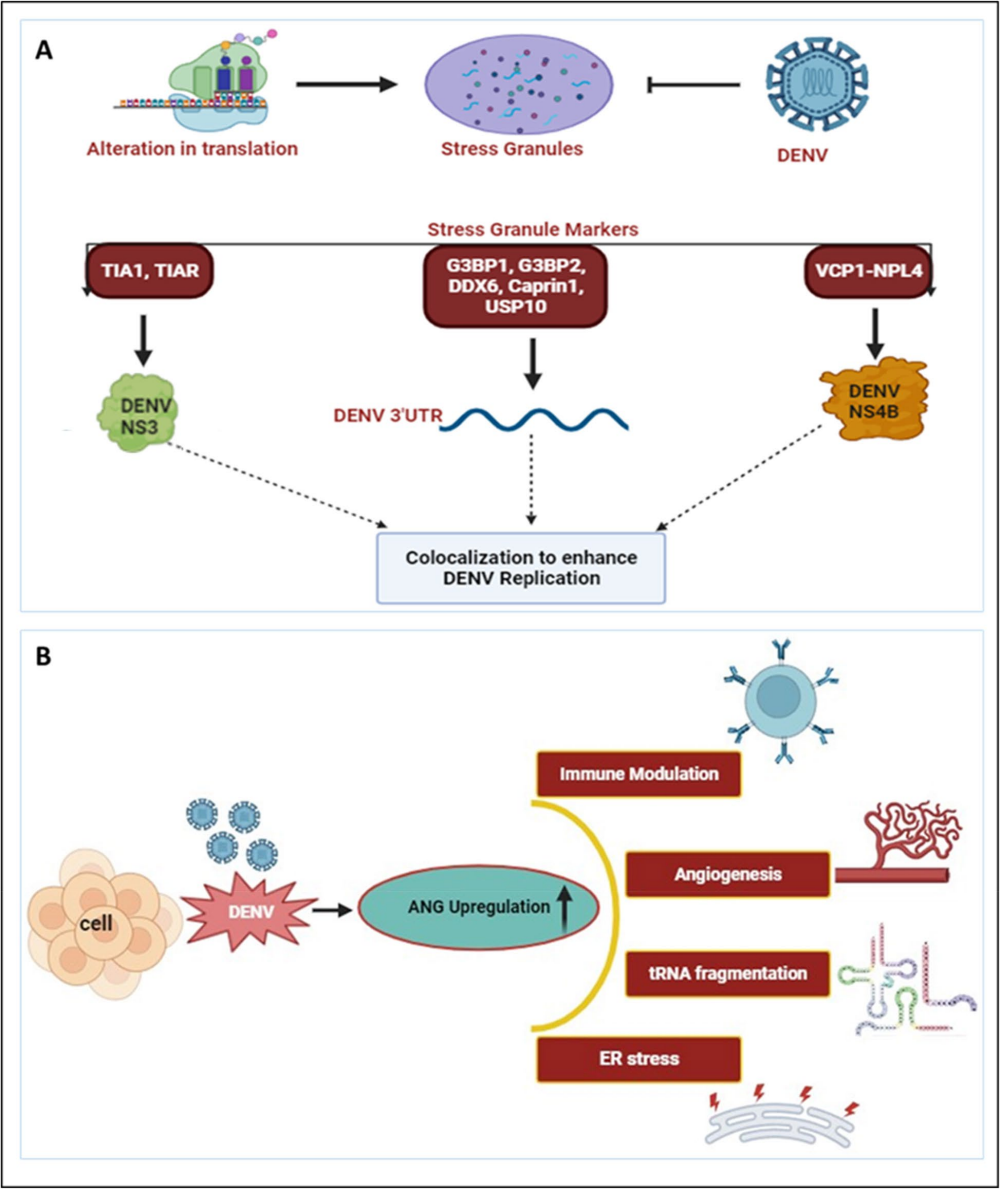
DENV and host stress responses: **A** Alterations in translation processes result in the assembly of stress granules. However, DENV prevents stress granule assembly by interacting with various stress granule markers. TIA1 and TIAR interact with DENV NS3, while various proteins such as G3BP1, G3BP2, DDX6, Caprin1, and USP10 bind to DENV 3’UTR. Additionally, VCP1, together with NPL4, forms a complex with DENV NS4B. These interactomes colocalize and participate in DENV replication [55–57]. **B** During DENV infection, Angiogenin levels have been found to be enhanced compared to uninfected controls. The upregulated Angiogenin may play a crucial role in various processes, including immune modulation, angiogenesis, tRNA fragmentation, and ER stress, among others [116]

Molecules such as T cell intracellular antigen (TIA1), TIA-1 related protein (TIAR), and Ras-Gap-SH3 domain-binding protein (G3BP) are among the biomarkers of SG, regulated during cellular stress [112, 113]. TIA1 and TIAR interact with DENV NS3, and colocalize in the perinuclear regions to enhance viral RNA formation and prevent SG formation, as shown in Fig. 7A. In contrast TIA1 and TIAR interacts with WNV 3’ (-) SL RNA to facilitate the virus replication [55]. Additionally, one study reported that DENV UTRs interact with host cellular proteins such as DDX6, G3BP1, G3BP2, Caprin-1, and USP10 as noted in Table 1 [56]. These proteins play roles in SG formation, but during DENV infection, they colocalize with DENV RNA, specifically interacting with DENV 3’UTR, significantly influencing DENV replication [56]. In contrast, G3BP1 and Caprin-1 form a stable complex with the capsid protein of ZIKV to benefit virus replication [114]. Furthermore, the capsid protein of JEV inhibits (arsenite-induced) SG formation by interacting with the SG protein Caprin-1 [115]. DENV employs various strategies to hinder SG assembly to enhance its survival. Valosin-containing protein (VCP), a cellular ATPase, reportedly plays a crucial role in DENV replication. VCP, together with Nuclear Protein Localization 4 (NPL4), forms a complex to enhance DENV propagation and disassembles stress granule formation. DENV NS4B activates VCP to evade stress response, preventing SG formation, while simultaneously facilitating cellular translation to enhance DENV replication and translation [57].

Several studies have indicated the modulation of RNases upon DENV infection. The activity of various ribonucleases (RNases) such as DICER, ELAC2, Angiogenin produced during host stress conditions, including virus infection, results in the formation of various regulatory RNAs [117]. Recently we have reported the synergistic correlation between host ribonuclease Angiogenin expression and DENV replication. Angiogenin (also known as RNase 5) is a member of the Ribonuclease A superfamily. Our finding revealed that, angiogenin levels are elevated during DENV infection. Interestingly, angiogenin levels remain unaffected by siRNA knockdown, suggesting that dengue infection may directly regulate angiogenin expression. As illustrated in Fig. 7B [116], upregulation of angiogenin during DENV infection may have a possible proviral effect, contributing to the replication and propagation of the dengue virus.

Dengue virus infection is known to induce endoplasmic reticulum (ER) stress, triggering signaling pathways associated with ER stress during viral infections. These pathways activate genes that support cell survival and anti-viral defenses. Viruses exploit the host’s cellular machinery, particularly the ER, to produce mature viral offspring and facilitate their replication. In response to the stress induced by alterations in protein production, the ER activates the Unfolded Protein Response (UPR) pathway to maintain ER homeostasis. Upon DENV infection, various UPR elements such as PERK, ATF6, and IRE1α are activated, which in turn activate innate immune factors, such as IRF3, PKR, and NF-κB. This approach might be employed to reduce viral infection. Thus, UPR components could serve as potential target of therapy for reducing DENV replication [118, 119].

DENV-NS1 alters cellular behaviour to increase the secretion of extracellular vesicles (EVs) containing miR-148a. These EVs are internalized by human microglial cells, where miR-148a interacts with the 3’UTR region of the ubiquitin-specific peptidase 33 (USP33) protein, leading to its downregulation. Consequently, reduced USP33 expression results in decreased levels of the activating transcription factor 3 (ATF3) protein. ATF3 plays a crucial role in regulating genes involved in proinflammatory pathways, such as TNF-α, NF-κB, and IFN-β. This study elucidates how DENV exploits the EV pathway to deliver miR-148a, thereby modulating USP33 and ATF3 levels in human microglial cells, ultimately contributing to inflammation within the Central Nervous System (CNS) [58].

## DENV and antiviral therapeutics

While there is currently no specific antiviral strategy approved to treat the dengue infection, ongoing research has explored various approaches to combat the infection. These strategies include investigating compounds with the potential to inhibit viral replication, immune modulators intended for enhancing the host’s immune response, and monoclonal antibodies designed to mimic the body’s natural defenses. Additionally, vaccine development has been a focus not only for prevention but also for potential therapeutic interventions. Several candidates are currently undergoing clinical trials as part of significant global efforts toward developing a preventive dengue vaccine. One of these, known as Dengvaxia, has been implemented and licensed in humans in several dengue-endemic countries. However, this vaccine has limitations, including approximately 60% efficacy and an association with antibody-dependent enhancement of disease severity in children, which impede its utilization. As of today, due to certain challenges, drug discovery efforts have not been able to provide any approved therapeutics to treat dengue virus infection [120].

**Fig. 8:**
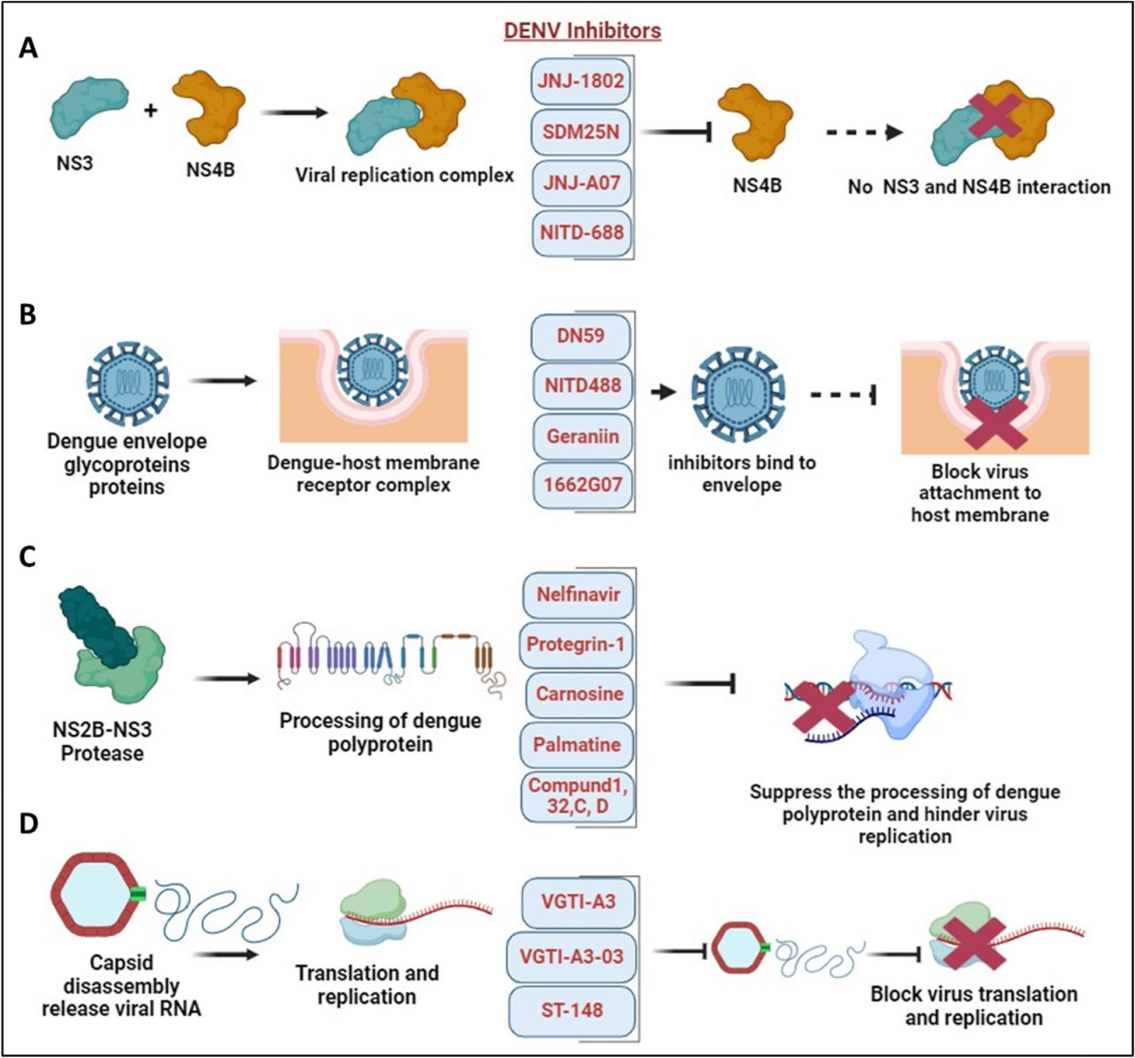
DENV and antiviral therapeutics: **A** Interaction between DENV NS3-NS4B in the formation of the virus replication complex. Several novel small molecule inhibitors, including JNJ-1802, JNJ-A07, SDM25N, and NITD-688, have been identified to inhibit NS4B, disrupting its interaction with NS3 and thereby suppressing DENV replication [122, 125, 128]. **B** DENV fusion inhibitors such as Geraniin, DN59, NITD-488, and 1662G07 bind with envelope protein thereby prohibiting virus attachment and entry into the host membrane [130–133]. **C** NS2B-NS3 protease complex participate in the processing of dengue polyprotein and supports virus replication. Inhibitors such as Nelfinavir, Protegrin-1, Carnosine, Palmatine, Compund 1, 32, C, D targets NS2B-NS3 protease complex and hinder virus replication [134–137]. **D** Dengue capsid protein undergoes capsid disassembly, releasing viral RNA for translation and replication. Inhibitors such as VGTI-A3, VGTI-A3-03, and ST-148 interact with the capsid protein, inducing antiviral effects and hindering dengue virus translation and replication [138, 139]

Recently, Obi JO et al. described a novel small molecule inhibitor, JNJ-1802. JNJ-1802 target the DENV NS4B protein and hinders the NS3 and NS4B interplay, as shown in Fig. 8A [121]. JNJ-1802 has demonstrated an effective barrier to resistance, *in-vitro* antiviral activity at low concentrations, and robust *in-vivo* efficacy in mice against four DENV serotypes. It has completed a phase I first-in-human clinical study in healthy individuals and was found to be safe [122]. Additionally, they also reported a compound, JNJ-A07, that exhibits antiviral activity against 21 clinical isolates of the dengue virus, spanning all known serotypes and genotypes of the dengue virus with a similar mechanism of action, but JNJ-1802 was selected over JNJ-A07 due to better preclinical safety profile [123]. Furthermore, several other DENV NS4B inhibitors have been developed, showing effectiveness as antivirals against dengue virus infection. Xie et al. demonstrated antiviral activity of NITD 618, with EC_50_ values ranging from 1.0 to 4.1 µM. This compound functions by inhibiting RNA synthesis, but its poor pharmacodynamics make it challenging for further development as a drug against DENV infections [124]. Similarly, SDM25N, an opioid receptor antagonist, was identified, and its activity also relies on NS4B, restricting RNA genome replication [125]. Another group also identified a similar compound, AM404, whose interactions with NS4B are yet to be validated [126]. Additionally, a spiropyrazolopyridone compound was detected that likely binds to valine 63 (V63) residue of NS4B present in DENV-2 and DENV-3, and restricts virus replication in the stated serotypes [127]. Hence, this candidate known as compound-14a was considered a potential preclinical contender for future development. Similarly, phenotypic screening of a Novartis compound library with 1.5 million compounds led to the identification of 13,000 potential inhibitors, selecting a hit, NITD-688, that displayed EC_50_ values of 8–38 nM inhibiting all DENV serotypes [128]. The compound’s interaction with NS4B was thoroughly characterized by NMR studies, demonstrating its potential for use in clinical research. Overall, these compounds validate DENV NS4B a validated target for the development of antivirals. It has been established that compounds or inhibitors that target DENV non-structural proteins interfere with viral replication and are possible targets for the development of antiviral treatments. Several drugs, namely Peptide 3, Peptide 4, Peptide 10, and Peptide 11, are involved in the inhibition of the NS1 protein as noted in Table 2 [129].

**Table 2:** Various anti-DENV therapeutics in development

**Target Proteins (DENV)**	**Compounds/ Inhibitors**	**References**
NS1	Peptide 3, Peptide 4, Peptide 10, and Peptide 11	[129]
NS3 Helicase	Ivermectin, ST-610, Suramin	[140–142]
NS2B-NS3 Protease	Protegrin-1, Retrocyclin-1, Nelfinavir, Carnosine, Palmatine, Thiazolidinone-peptide hybrids, Compound 32, Compound 1, 166347, ARDP0006, ARDP0009, Compound 7n, Diaryl(thio)ethers, Compound C, and Compound D	[134–137]
NS4B	JNJ-1802, JNJ-A07, NITD 618, SDM25N, AM404, and NITD-688	[122–126, 128]
Envelope	1662G07, DN59, NITD488, and Geraniin	[130–133]
Membrane	MLH40	[143]
Capsid	VGTI-A3, VGTI-A3-03, ST-148	[138, 139]

In the pursuit of effective therapeutics against DENV, researchers have targeted specific viral proteins crucial for its replication within host cells. Among these targets, the NS3 helicase and NS2B-NS3 protease have garnered considerable attention due to their essential roles in viral replication. Ivermectin, a drug well known for its antiparasitic properties, has emerged as a promising candidate for DENV treatment. Suputtamongkol et al. [140] reported on a phase 2/3 clinical trial investigating Ivermectin’s efficacy, revealing its ability to suppress NS3 helicase and NS2B-NS3 protease activity, consequently hindering DENV replication in host cells as mentioned in Table 3. Various compounds have been explored for their potential to inhibit NS3-helicase and NS2B-NS3 protease activity, thereby disrupting viral replication. Lee et al. [137] outlined several promising candidates, including ST-610 [141] and Suramin [142] as NS3-helicase inhibitors, alongside a range of compounds targeting NS2B-NS3 protease such as Protegrin-1 [134], Retrocyclin-1 [135], Nelfinavir, Carnosine, Palmatine, and Thiazolidinone-peptide hybrids. Additionally, compounds like Compound 32, Compound 1, 166347, ARDP0006, ARDP0009, Compound 7n, Diaryl(thio)ethers, Compound C, and Compound D have shown potential in inhibiting NS2B-NS3 protease activity [136, 137].

**Table 3:** DENV antivirals that are currently undergoing clinical trials

**Drug Name**	**Target**	**Clinical Data**	**References**
JNJ-1802	NS4B inhibitor	Phase- 2 (NCT05201794)	[122]
UV-4B	Inhibits endogenous alpha glucosidase	Phase 1a clinical trial (NCT02061358)	[144]
AT-752	Inhibits NS5 RdRp function	Phase 1 (NCT05366439) and phase 2(NCT05466240) clinical trials are in progress	[145]
Zanamivir	Blocks desialylation on platelet membrane by acting as neuraminidase inhibitor	Early Phase 1 (NCT04597437)	[146]
Ivermectin	Inhibits the host nuclear import proteins important for nuclear exchange of the dengue NS5 protein	Phase 2/ 3 (NCT02045069)	[140]
Ketotifen	Prevent mast cell degranulation	Phase 4 (NCT02673840)	[147]

In addition to this, several drugs are designed to target structural proteins, as described in Table 2. For instance, 1662G07 and some analogs target the envelope protein as DENV fusion inhibitors [130]. Likewise, DN59 [131], and NITD448 [132] similarly inhibit the E-protein. Compound like Geraniin target the E-protein to inhibit virus binding and entry as shown in Fig. 8B [133]. Furthermore, MLH40 inhibits the interplay between the envelope and the membrane proteins by specifically targeting the prM/M protein [143]. VGTI-A3 and VGTI-A3-03, by binding with the capsid protein, hinder the interplay between the DENV C protein and host intracellular lipid droplet, thereby disrupting the binding and thus prohibiting virus entry into cells [138]. A study by Xia et al. demonstrated the effect of a novel compound, ST-148, that inhibits the capsid protein of the dengue virus and interferes in virus replication as mentioned in Fig. 8D. ST-148 interacts with the α1-α1′ helices region of the capsid protein, resulting in reduced viremia as well as low cytokine levels in the plasma of DENV-infected AG129 mouse model. One structural approach showed that ST-148 promotes two capsid dimers to engage in a “kissing” interaction [139]. However, a recent investigation reported that ST-148 inhibits DENV-2 but not DENV-1, DENV-3, and DENV-4. The antiviral and resistance mechanisms of ST-148 are yet unclear [148]. Pre-clinical data of some antiviral agents in the advance stage have shown potential effect against DENV. AT-752, an inhibitor of NS5 RdRp function, is in Phase 2 clinical trial as mentioned in Table 3 [145]. It reduced the viremia in immunocompromised mouse models infected with DENV. Ketotifen, a drug in Phase 4 clinical trial, reduced vascular leakage in a mouse model infected with DENV [147]. Zanamivir, and UV-4B as mentioned in Table 3 are still in the early phase of clinical trial [144, 146]. Although many of these molecules and compounds are promising candidates for further development, significant limitations of these antivirals involve cytotoxicity and adverse effects. Moreover, clinical trials are crucial for evaluating the safety and efficacy of these antiviral therapies, and their success holds promise for advancing the treatment options available for dengue patients.

## Conclusion

The root cause and progression of the disease are significantly influenced by the complex molecular interactions between the dengue virus and the host cellular machinery. In this review, we delve into the interplay between DENV and the host molecular machinery, focusing on epigenetic regulations, transcription, post-transcription, translation, and post-translation processes, including stress granules. These factors play a crucial role in shaping the evolution of an infection, determining the severity of symptoms, and influencing the host body’s defense system response. Epigenetic alterations such as DNA methylation, histone modifications, and involvement of various non-coding RNAs regulate DENV replication, immunological responses, and disease development. The virus exploits these alterations, manipulating host cellular components to facilitate its proliferation and evade immune responses. Transcriptional regulation is another essential aspect of dengue infection, as the virus manipulates the host’s transcriptional machinery to support its development and hinder immune responses. By appropriating key transcription factors and regulators, DENV modifies gene expression patterns, impacting both immune responses and viral pathogenesis.

Similarly, DENV exerts control over its own gene expression and alters the host cell environment through modulating post-transcriptional modifications, including alternative splicing and RNA degradation. Furthermore, at the translational level, DENV competes with host translation factors, employing techniques such as internal ribosome entry sites (IRES) to initiate translation and ensure efficient protein synthesis. Post-translational changes of both DENV and host proteins also play a role in viral replication, immune evasion, and host response regulation. Understanding these complex interactions at the molecular level is imperative for developing efficient diagnostic, therapeutic, and preventive measures against dengue virus infection. Targeting specific elements within these regulatory networks may open new avenues for therapeutic development and intervention, ultimately reducing the impact of this pervasive and often severe dengue infection.

## Data Availability

Not applicable.

## Abbreviations

DF: Dengue fever
DHF: Dengue hemorrhagic fever
DSS: Dengue shock syndrome
NSAID: Non-steroidal anti-inflammatory medicines
ADE: Antibody-dependent enhancement
ADCC: Antibody-dependent cellular cytotoxicity
CS: Complement system
UTR: Untranslated Region
CDK8: Cyclin dependent kinase 8
HK2: Hexokinase 2
CCR10: C-C Motif Chemokine Receptor 10
PLXNA3: Plexin-A3
CTBP1-AS1: C-terminal-binding protein antisense 1
MAFG-AS1: MAF BZIP transcription factor G antisense RNA 1
IFN-γ: Interferon gamma
TNFα: Tumour necrosis factor α
CTCF: CCCTC‐binding factor
P-TEFb: Positive transcription elongation factor b
NF-κB: Nuclear factor kappa B
IL-8: Interleukin-8
TAL-1: T-Cell Acute Lymphocytic Leukemia 1
snRNPs: Small nuclear ribonucleoproteins
CD2BP2: CD2 cytoplasmic tail binding protein 2
DDX23: DEAD box helicase 23
XRN1: 5’-3’ Exoribonuclease 1 protein
RIG-I: Retinoic acid-inducible gene I
IKKϵ: Inhibitor of nuclear factor kappa-B kinase ε
DEGs: Differentially expressed genes (DEGs)
RBPs: RNA-binding proteins
hnRNPs: Heterogeneous nuclear ribonucleoproteins
sfRNA: Sub genomic flavivirus RNA
ISGs: Interferon-stimulated genes
VSR: Viral Suppressors of RNA Silencing
HSPA1A: Heat shock protein family A (Hsp70) member 1A
MOV10: MOV10-RISC complex RNA helicase
UPF1: Upstream frameshift 1
vgRNA: Viral genomic RNA
RNAi: RNA Interference
eIF4E: Eukaryotic translation initiation factor 4E
G3BP1: G3BP Stress Granule Assembly Factor 1
G3BP2: G3BP Stress Granule Assembly Factor 2
CBC: Cap binding complex
IRES: Internal ribosomal entry site
PTMs: Post-translational modifications
TRIM: Tripartite motif family members
cGAS: Cyclic GMP-AMP synthase
STING: Stimulator of interferon genes
DG-SIGN: Dendritic cell specific ICAM3 grabbing non-integrin receptor
MBL: Mannose binding lectin
STAT2: Signal transducer and activator of transcription 2
HDAC: Histone deacetylase
DNMT: DNA methyltransferase
DIDO1: Death inducer obligator 1
SGs: Stress granules
TIA1: T cell intracellular antigen
TIAR: TIA-1 related protein
G3BP: Ras-Gap SH3 domain binding protein
USP10: Ubiquitin Specific Peptidase 10
VCP: Valosin containing protein
NPL4: Nuclear protein localization 4
EDI: Extradomain A of fibronectin
Bclx: B-cell lymphoma-extra-large
CFTR: Cystic fibrosis transmembrane regulator
RBM10: RNA-binding protein 10
RIG-I: Retinoic acid-inducible gene I
HuR: Hu Antigen R
LARP1: La- Related Protein 1
PABPC: Cytoplasmic poly(A)-binding protein
PAIP2: Poly(A) Binding Protein Interacting Protein 2
PERK: PKR-like ER kinase
ATF6: Activating transcription factor 6
ATF3: Activating transcription factor 3
IRE1α: Inositol-requiring enzyme 1 alpha
PKR: Protein kinase R
IRF3: Interferon regulatory transcription factor 3
USP33: Ubiquitin carboxyl-terminal hydrolase 33
